# Ibrutinib Treatment of Mantle Cell Lymphoma Relapsing at Central Nervous System: A Case Report and Literature Review

**DOI:** 10.1155/2017/9583257

**Published:** 2017-07-16

**Authors:** Donato Mannina, Barbara Loteta

**Affiliations:** Hematology Unit, Papardo Hospital, c/da Papardo, 98158 Messina, Italy

## Abstract

Mantle cell lymphoma (MCL) accounts for about 5% of all lymphomas. Its clinical and histological features are heterogeneous. After a frequently good initial response, the disease generally and repeatedly relapses and finally the outcome is poor. Particularly severe is the prognosis of the rare occurrence of CNSi (Central Nervous System involvement). Ibrutinib, an oral inhibitor of Bruton tyrosine kinase (BTK), has shown strong activity in relapsing patients with Chronic Lymphocytic Leukemia (CLL) and MCL. Few reports are available about treatment with ibrutinib of patients presenting CNSi by lymphoproliferative diseases (LPD). In all of them, ibrutinib, at the dosage between 420 and 560 mg/day, showed an impressive effectiveness. Here we describe a case of MCL with CNS relapse showing an excellent response to ibrutinib administered at the unusual dose of 280 mg/day because of concomitant treatment of cardiological disease.

## 1. Introduction

Mantle cell lymphoma (MCL) is a subtype of B-cell non-Hodgkin's lymphoma (NHL) accounting for about 5% of all lymphomas [1]. Lymph node or extranodal biopsy is essential for diagnosis. Positivity of CD5 and CD19 and negativity of CD23 are the immunohistochemical markers. Cytogenetic and molecular findings are the translocation t(11;14)(q13:q32) and the overexpression of Cyclin D1.

The clinical presentation is heterogeneous.

The majority of the patients is in advanced stage at presentation and may have extranodal involvement. The response at first-line therapy is generally good. Unfortunately, most of the patients eventually relapse and the outcome after salvage treatment is very disappointing with a median overall survival of about three years.

First-line therapy consists generally of ARA-C containing regimens followed by autologous hemopoietic stem cells transplantation (HSCT).

The central nervous system (CNS) involvement is a relatively rare event in advanced/relapsed MCL [2, 3]. This is a very severe condition with a very poor prognosis.

Ibrutinib is an oral inhibitor of Bruton tyrosine kinase (BTK) recently approved for relapsing/refractory (R/R) patients with Chronic Lymphocytic Leukemia (CLL) and MCL [4, 5].

We report a case of MCL with CNS relapse that showed an excellent response to ibrutinib.

## 2. Case Report

A 59-year-old man was diagnosed with classical MCL at another institution in April 2012.

At diagnosis, ECOG performance status was one, Ann Arbor stage was IV for bone marrow and gastric and intestinal involvement. Axillary, laterocervical, supraclavear, mediastinal, celiac, crural, iliac, inguinal, paralumboaortic, and perigastric lymph nodes were enlarged. The disease presented with leukemic expression. No B symptoms were present. MIPI score was five.

The patient was treated according to MCL0208 protocol designed by the Fondazione Italiana Linfomi (FIL), consisting in an induction phase (3 cycles of R-CHOP, given every 21 days) followed by a consolidation phase (high-dose cyclophosphamide, 2 cycles of high-dose Ara-C, BEAM, and HSCT). After completion of first-line high-dose chemotherapy, patients achieving complete or partial response went to randomization between maintenance with lenalidomide or observation.

Our patient, randomized for maintenance, came to our observation in complete remission (CR) on December 2013, after 6 cycles of the maintenance phase. At our institution, the maintenance program was continued and finally completed on Jun 2015.

During the follow-up, the patient was continuously in CR.

In March 2016, the admission at an Orthopedic Unit was necessary, due to severe back pain resulting from a fall that had caused a spinal fracture and a fracture of the left wrist. A few days after the discharge, a new hospitalization at a Neurological Department occurred urgently because of aphasia and mental obnubilation. Brain CT scan was negative for focal lesions; however, the lumbar puncture (LB) showed the presence of 300 mononucleated cells (MNC)/*µ*l in the cerebrospinal fluid (CSF). The patient came to our institution with the suspected recurrence of lymphoma at CNS.

At admission, the patient's general conditions were poor. He was bedridden. His mental state was compromised and erratically shiny. Arterial pressure was 140/80 mm/Hg, heart rate 72/m′, SpO2 96%, and *T* 36,7°C. Peripheral blood count and biochemistry tests were normal except for light increase of sALT. Whole-body CT scan and trephine biopsy were negative so systemic relapse was excluded.

**Figure 1:**
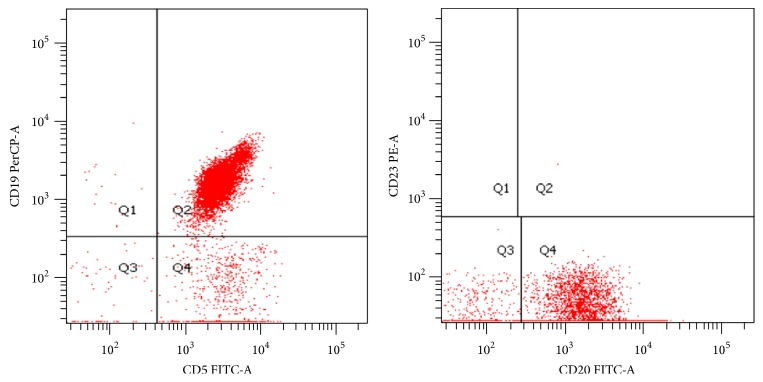
Flow cytometry on CSF reveals a positive expression of CD19, CD20, and CD5 and negativity of CD23.

We performed a first LP with methotrexate (MTX) 12 mg. It showed the presence of 800 MNC/mm^3^. The immunophenotype showed the typical pattern of mantle cell lymphoma (CD5/19+, CD23 neg) (Figure 1).

We performed further LP with MTX (12 mg) and ARA-C (50 mg) two times a week for six times, until the cell count in the CSF fell to 5 cells/*µ*l. The clinical condition of the patient gradually improved. We discharged the patient on April 19, after 20 days from hospitalization.

We started ibrutinib at the dose of 280 mg/day. We used this dosage because of a history of repeated episodes of atrial fibrillation and ongoing prophylactic treatment with oral amiodarone. The patient was not on anticoagulation therapy. No other LPs were performed nor any systemic chemotherapy was administrated.

In December 2016, the patient came to clinical control complaining of back pain without other symptoms. We stopped ibrutinib administration because of the potential bleeding risk related to invasive procedures and, 2 days after, we performed a LP. No cells were present in CSF thus excluding the suspicion of a new relapse. The back pain was very likely due to concomitant discopathy. Then we restarted ibrutinib administration.

Currently, 13 months after starting ibrutinib, the patient continues to be in CR.

## 3. Discussion

The involvement of CNS by a relapsing MCL is a very serious condition with high mortality rate and a median overall survival of approximately 3,7 months [2].

Very few reports are available in literature, but the incidence of CNS involvement in MCL might be higher than previously recognized.

In a study of 1994, of 22 patients with MCL, 6 developed CNS involvement at a median of 18 months from diagnosis. All of them had poor MCL histological subtypes and advanced disease. In most of the cases, CNS infiltration was part of resistant disease or generalized relapse and had an ominous significance [6].

Ibrutinib has shown a remarkable efficacy in R/R MCL with a 68% of overall response rate (ORR) as single agent [7–9].

Few data are available, at the time, concerning the efficacy of ibrutinib in the treatment of CNSi in LPD (Table 1). Previous pharmacokinetics studies have documented the drug's ability to cross the blood-brain barrier [10]. This phenomenon is not restricted to MCL, and clinical CNS responses can occur at the lower ibrutinib dose of 420 mg/day as in CLL.

In a recent report, three patients with CNS relapse of MCL received ibrutinib at full dose of 560 mg/day [11], with obtainment of a CR during 2 months to a year.

A series of 5 cases with systemic MCL involving the CNS was collected from 4 centers in the UK, where they were treated with ibrutinib (560 mg q.d.) as single agent or in combination with high-dose antimetabolites or steroids alone. One patient received additional intrathecal cytarabine. With a median follow-up of 4 months, the ORR rate was 100%. The median time to clinical response was 2 weeks. At the time of writing, the median duration of response was 4 months and two patients were in clinical remission. Toxicity from ibrutinib therapy was minimal. No significant additional toxicity or bleeding events occurred [12].

Three recent papers concern ibrutinib treatment of CNS involvement in LPD other than MCL.

Wanquet et al., on behalf of the French Innovative Leukemia Organization [13], reported a series of 4 consecutive cases of CNSi in CLL treated with ibrutinib monotherapy at the dose of 420 mg/day.

An Australian group [14] favorably treated another case of CNS involvement in B-CLL.

Finally, two cases of Waldenstrom Macroglobulinemia (WM) complicated by Bing-Neel Syndrome (BNS) were successfully managed with ibrutinib at the same 420 mg/day dose, after a transient neurological improvement achieved with R-HDAC [15].

The case of MCL we illustrated here was treated with a lower dose. We decided this dose (280/mg/day) both for the aforementioned arrhythmogenic risk of this patient and for concomitant use of amiodarone which, as is well known, may increase ibrutinib serum levels due to competitive activity upon CYP3A4 [16, 17].

Additionally, except for the six therapeutic LP initially administrated, we used ibrutinib as single agent. Nevertheless, the drug showed an impressive effectiveness in maintaining CR and preventing relapse, also demonstrating the ability to cross blood-brain barrier at this dosage. That is noteworthy, especially in a very serious and prognostically unfavorable condition lacking efficacious therapeutic alternatives for the long-term control of the disease.

This result could suggest modulating the standard dose in some cases, reducing both off-target effects and drug-related complications and allowing treating a larger number of patients.

## Figures and Tables

**Table 1:** Review of ibrutinib therapy for patients with LPD and CNS involvement.

Ref.	Age	Sex	Diagnosis	Prior lines	CNS therapy	FU	Response	DOR	Toxicity
[11]	61	M	MCL	3	Ibrutinib 560 mg	12 m	CR	Ongoing	None
[11]	62	M	MCL	2	Ibrutinib 560 mg	9 m	CR	Ongoing	None
[11]	77	F	MCL	2	Ibrutinib 560 mg	2 m	PR	Ongoing	None
[12]	54	M	MCL	1	HD-MTX and HiDAC + Ibrutinib 560 mg/d	4 m	PR	4 m	Bruising
[12]	55	M	MCL	1	Ibrutinib 560 mg MP 500 mg 4 d	5 m	PR	4 m	None
[12]	65	M	MCL	1	Ibrutinib 560 mg Dexamethasone IT Cytarabine	4 m	CR	Ongoing	None
[12]	58	M	MCL	1	Ibrutinib 560 mg	5 m	PR	Ongoing	None
[12]	57	M	MCL	1	HD-MTX Ibrutinib 560 mg	1 w	Transient PR	6 d	None
[13]	58	M	CLL	8	HiDAC, MTX, oxaliplatin, R- monotherapy, IT-CT, Iv immunoglobulins → Ibrutinib 420 mg/d	9 m	CR	9 m	Atrial fibrillation → death (stroke)
[13]	65	M	CLL	4		14 m	CR	Ongoing	None
[13]	63	M	CLL	2		8 m	CR	Ongoing	None
[13]	68	F	CLL	0		9 m	CR	Ongoing	None
[14]	66	M	CLL	0	Ibrutinib 420 mg/d	8 m	CR	Ongoing	None
[15]	72	M	WM/BNS	1	R, HD-MTX IT liposomal cytarabine → Ibrutinib 420 mg/d	6 m	CR	Ongoing	None
[15]	56	M	WM/BNS	1	R-HiDAC → Ibrutinib	6 m	CR	Ongoing	None

DOR: duration of response; MP: methylprednisolone; HD-MTX: high-dose methotrexate; HiDAC: high-dose ARA-C; IT-CT: intrathecal chemotherapy; m: months; w: week; d: days.
